# Phosphoinositide signaling at the cytoskeleton in the regulation of cell dynamics

**DOI:** 10.1038/s41419-025-07616-x

**Published:** 2025-04-14

**Authors:** Xiaoting Hou, Yu Chen, Noah D. Carrillo, Vincent L. Cryns, Richard A. Anderson, Jichao Sun, Songlin Wang, Mo Chen

**Affiliations:** 1 Department of Pharmacology, Joint Laboratory of Guangdong-Hong Kong Universities for Vascular Homeostasis and Diseases, SUSTech Homeostatic Medicine Institute, School of Medicine, Southern University of Science and Technology, Shenzhen, China; 2 Department of Medicine, School of Medicine and Public Health, University of Wisconsin-Madison, Madison, WI USA; 3 University of Wisconsin Carbone Cancer Center, School of Medicine and Public Health, University of Wisconsin-Madison, Madison, WI USA; 4 Department of Critical Care Medicine, Shenzhen People’s Hospital (The Second Clinical Medical College, Jinan University; The First Affiliated Hospital, Southern University of Science and Technology), Shenzhen, China; 5 Guangdong Provincial Clinical Research Center for Geriatrics, Shenzhen Clinical Research Center for Geriatrics, Shenzhen People’s Hospital, Shenzhen, China; 6 Beijing Laboratory of Oral Health, Capital Medical University, Beijing, China

**Keywords:** Actin, Lipid signalling

## Abstract

The cytoskeleton, composed of microfilaments, intermediate filaments, and microtubules, provides the structural basis for cellular functions such as motility and adhesion. Equally crucial, phosphoinositide (PIP_n_) signaling is a critical regulator of these processes and other biological activities, though its precise impact on cytoskeletal dynamics has yet to be systematically investigated. This review explores the complex interplay between PIP_n_ signaling and the cytoskeleton, detailing how PIP_n_ modulates the dynamics of actin, intermediate filaments, and microtubules to shape cellular behavior. Dysregulation of PIP_n_ signaling is implicated in various diseases, including cancer, highlighting promising therapeutic opportunities through targeted modulation of these pathways. Future research should aim to elucidate the intricate molecular interactions and broader cellular responses to PIP_n_ signaling perturbations, particularly in disease contexts, to devise effective strategies for restoring cytoskeletal integrity.

## Facts

- The molecular mechanisms underlying the direct interaction between PIP_n_s and intermediate filaments remain poorly defined despite evidence of their role in regulating IF dynamics.
- PIP_n_ signaling exhibits spatial and temporal regulation across cytoskeletal components, raising key questions about signal coordination during processes like migration and division.
- The therapeutic potential of targeting PIP_n_-cytoskeleton interactions remains largely unexplored despite their crucial roles in cellular dynamics and disease pathogenesis.

## Open questions

- What mechanisms enable PIP_n_s to specifically recognize and target distinct cytoskeletal components, and how is this selectivity determined?
- What are the full sets of PIP_n_-binding proteins involved in cytoskeletal regulation, and how do their functions vary in different cellular contexts?
- How do PIP_n_ signaling pathways integrate with other networks to orchestrate cytoskeletal dynamics during development, homeostasis, and disease?

## Introduction

The cell cytoskeleton is a complex network of protein filaments that extends throughout the cell. It provides structural support and plays a dynamic role in cellular processes such as motility, division, and intracellular transport. The cytoskeleton comprises three main types of protein polymers: microfilaments, intermediate filaments (IFs), and microtubules, each with distinct roles and properties [1].

**Fig. 1:**
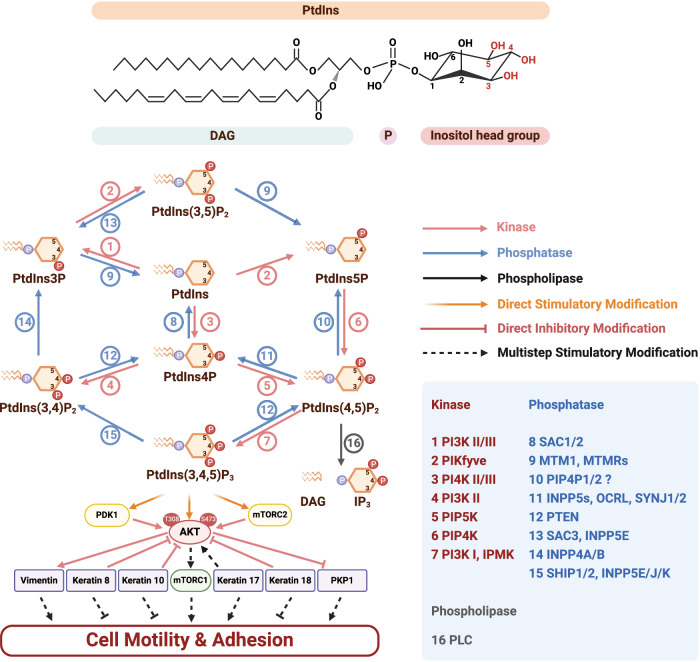
Overview of PI metabolism and the PI3K/AKT signaling pathway in cell dynamics. This diagram provides a comprehensive overview of PI metabolism within the cellular environment, highlighting the structure of the inositol ring with the 3rd, 4th, and 5th hydroxyl groups capable of phosphorylation. This process generates seven distinct PIP_n_ isomers, whose interconversion is facilitated by PIP_n_ kinases, phosphatases, and phospholipases—critical enzymes in this metabolic pathway. The diagram also highlights the PI3K/AKT signaling pathway, a key downstream effector of PIP_n_ metabolism that regulates various cellular processes, particularly cell dynamics such as motility and adhesion. Notably, PtdIns(4,5)P_2_ 4-phosphatase type I/II (PIP4P1/2), also known as TMEM55B/TMEM55A, has been proposed as PtdIns(4,5)P_2_ 4-phosphatase [172]. However, the follow-up study found PIP4P1/TMEM55B to be catalytically inactive and lacking the typical catalytic CX_5_R motif [173]. To reflect this controversy, PIP4P1/2 is accompanied by a question mark, denoting the ongoing debate in the field. This diagram is generated using BioRender.

Phosphoinositide (PIP_n_) signaling is a pivotal regulatory mechanism that modulates cytoskeletal dynamics. Phosphatidylinositol (PI/PtdIns), which consists of a glycerol backbone, two fatty acid acyl chains, and a *myo*-inositol ring, undergoes reversible phosphorylation at the 3rd, 4th, and 5th positions of the *myo*-inositol ring by specific kinases and phosphatases (Fig. 1) [2]. This phosphorylation produces seven unique PIP_n_ isomers, including three monophosphorylated forms (PtdIns3P, PtdIns4P, and PtdIns5P), three bisphosphorylated forms (PtdIns(3,4)P_2_, PtdIns(3,5)P_2_, and PtdIns(4,5)P_2_), and one triphosphorylated form (PtdIns(3,4,5)P_3_). These PIP_n_s serve as key signaling molecules that influence a plethora of cellular processes.

Discovered in the 1950s by Lowell and Mabel Hokin, the PIP_n_ signaling cycle has since been recognized as a pivotal pathway in biology despite PIs representing a minor component of cellular membranes [3]. PIP_n_s are present in the plasma membrane, Golgi apparatus, endoplasmic reticulum, endosomes, lysosomes, and the nuclear envelope [4]. Early studies focused on the biochemical and functional roles of membrane-associated PIP_n_s, but research in the 1960s and 1970s revealed phospholipids within the nuclear matrix and chromatin [5, 6]. In 1983, Smith and Wells detected PI kinase and PIP kinase activity in the nucleus [7], leading to Cocco et al.‘s discovery in 1987 of nuclear PIP_n_ generation [8]. These discoveries expanded the scope of membrane-bound PIP_n_ signaling into non-membranous regions within the nucleus. Over the past 30 years, extensive evidence has demonstrated that PIP_n_s also localize in distinct non-membranous subcellular compartments, including the nucleoplasm, nuclear matrix, nucleolus, nuclear speckles, nuclear PtdIns(4,5)P_2_ islets, chromatin, ribosomes, centrosomes, cytoskeleton, and inflammasomes, with specific PI transfer proteins (PITPs), lipid kinases and phosphatases regulating these regional PIP_n_ pools [2, 9–17]. The cytoskeleton is one such non-membranous compartment where PIP_n_ signaling not only regulates cytoskeletal dynamics but also provides a link with cellular membranes [18].

In the context of cell dynamics, PIP_n_ signaling interfaces with the cytoskeleton to orchestrate the formation and retraction of cellular protrusions, such as lamellipodia and filopodia, which are primarily driven by the polymerization and depolymerization of actin microfilaments [18–20]. The coordinated action of PIP_n_ signaling and the cytoskeleton is essential for cell polarization, directional movement, and the establishment of stable adhesion sites with the extracellular matrix or neighboring cells. This review aims to dissect the intricate relationship between PIP_n_ signaling and the cytoskeleton, focusing on the role of PIP_n_s in regulating the dynamics and function of microfilaments, IFs, and microtubules in cell motility and adhesion. We will explore the molecular mechanisms by which PIP_n_ signaling influences cytoskeletal remodeling and the consequences of these interactions for cellular operations.

## PIP_n_ signaling at microfilaments

Microfilaments, also known as actin filaments, are essential cytoskeleton components with a diameter of ~7 nm [21]. These thin, flexible filaments comprise actin monomers (G-actin) that polymerize into filamentous actin (F-actin) in a highly regulated process [22, 23]. Actin filaments are inherently polarized, with a fast-growing barbed (+) end and a slower-growing pointed (−) end [22, 23]. The polarity of microfilaments is central to their dynamic behavior; ATP-bound G-actin monomers are preferentially added to the barbed end, promoting filament elongation, while ADP-bound G-actin dissociates from the pointed end [23, 24]. This dynamic process, known as treadmilling, allows for the continuous turnover of actin filaments with an evident influence on processes like cell motility, shape changes, and intracellular transport [25].

Functionally, microfilaments are crucial for maintaining cell shape and providing mechanical support through the actin cortex beneath the plasma membrane [21, 23]. They facilitate cell motility by driving the extension of lamellipodia and filopodia, which are essential for migration [1, 26, 27]. During cytokinesis, microfilaments form a contractile ring that constricts the cell membrane; however, the process also involves complex membrane trafficking from endosomes and a tightly regulated abscission phase to complete cell division. They also serve as tracks for myosin motor proteins to transport vesicles and organelles [24, 28, 29]. Also, microfilaments are critical in signal transduction, linking extracellular signals to intracellular responses by interacting with membrane receptors and influencing cellular responses [24, 30]. Furthermore, microfilaments are crucial in cell adhesion, connecting cells to the extracellular matrix and each other via structures like focal adhesions (FAs). These functions underscore the importance of microfilaments in inter- and intra-cellular dynamics, in which PIP_n_ signaling pathways play a significant role.

The rapid polymerization and depolymerization of microfilaments enable cells to respond efficiently to environmental cues, especially during cell migration, where actin filaments form protrusions such as lamellipodia and filopodia at the leading edge [31–33]. Additionally, actin filaments provide mechanical support to the cell cortex, maintaining cell integrity and facilitating processes like cytokinesis [33, 34]. The interaction of actin with various actin-binding proteins, including profilin, cofilin, and actin-related proteins-2/3 (ARP2/3), further regulates filament dynamics and architecture, allowing cells to execute complex mechanical tasks, such as membrane deformation, vesicle transport, and contractile ring formation during cell division [31, 33, 35, 36].

The actin cytoskeleton is sculpted by many actin-binding proteins (ABPs) that bind, twist, sever, branch, or cap actin filaments (e.g., cofilin, gelsolin, profilin, etc.) [35, 36]. The activities of ABPs are regulated by PIP_n_s, which in turn influence cellular activities [22, 23, 37, 38]. PIP_n_ signaling is integral to regulating actin dynamics, mainly through the actions of PtdIns(4,5)P_2_ and PtdIns(3,4,5)P_3_ in conjunction with ABPs [36, 39].

**Fig. 2:**
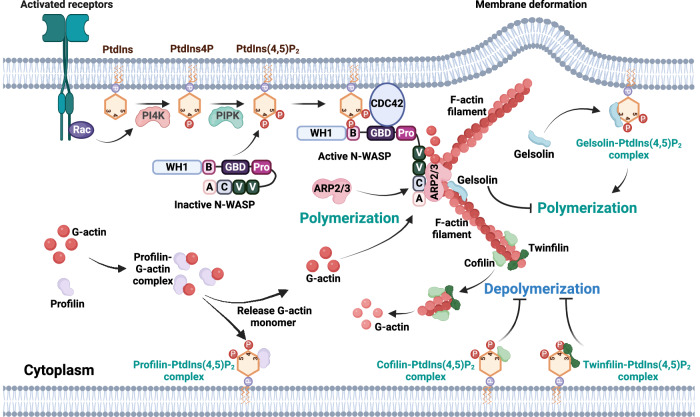
PIP_n_ regulation of actin dynamics via actin-binding proteins (ABPs). This figure illustrates the critical role of PtdIns(4,5)P_2_ in regulating actin filament assembly and disassembly through its interaction with various ABPs. At the plasma membrane, PtdIns(4,5)P_2_ interacts with ABPs such as cofilin and twinfilin, which promote filament depolymerization, while binding with profilin enhances actin polymerization. Cofilin and twinfilin are sequestered by PtdIns(4,5)P_2_, preventing filament disassembly, while profilin-G-actin complexes are regulated by PtdIns(4,5)P_2_ to control actin monomer availability. N-WASP contains a WASP Homology 1 domain (WH1) that binds WIP-family proteins, a basic sequence for PtdIns(4,5)P_2_ binding (B), a GTPase-binding domain (GBD) that interacts with CDC42-GTP, a proline-rich region (Pro), and a VCA domain, which consists of a V (verprolin homology) region for actin binding, a C (central) domain, and an A (acidic) motif that binds the ARP2/3 complex to promote actin nucleation. The interaction of N-WASP with PtdIns(4,5)P_2_ and CDC42 leads to the activation of ARP2/3 and subsequent actin polymerization. Additionally, PtdIns(4,5)P_2_ modulates the activity of gelsolin, inhibiting its filament-severing functions, thereby maintaining actin filament stability under dynamic conditions of cellular movement and membrane deformation. This interplay is critical for processes such as cell motility and structural organization. This diagram is generated using BioRender.

PtdIns(4,5)P_2_ is a critical PIP_n_ located at the plasma membrane that modulates actin dynamics by interacting with a range of ABPs [20, 39–42]. PtdIns(4,5)P_2_ generally inhibits ABPs that promote actin filament disassembly and activates those that enhance actin polymerization. Consequently, PtdIns(4,5)P_2_ facilitates the formation of actin structures, particularly under the plasma membrane and around organelles enriched with PIP_n_s (Fig. 2).

For instance, cofilin, a protein responsible for actin filament depolymerization, binds to ADP-actin filaments to promote their disassembly [1]. Competitively, PtdIns(4,5)P_2_ sequesters cofilin, preventing its interaction with actin, thus stabilizing the filaments [42–44]. Upon a decrease in PtdIns(4,5)P_2_ levels, cofilin is released, leading to heightened filament turnover and disassembly. Profilin, another actin-binding protein, facilitates actin polymerization by binding G-actin and promoting the exchange of ADP for ATP [22, 45, 46]. PtdIns(4,5)P_2_ interacts with profilin and regulates its availability, thereby controlling the pool of actin monomers available for filament formation [47, 48]. PtdIns(4,5)P_2_ suffices to disrupt the actin-profilin complex and inhibit profilin’s actin monomer sequestration activity. Through this mechanism, PtdIns(4,5)P_2_ helps regulate the rate of actin polymerization. Like cofilin, changes in the concentration of PtdIns(4,5)P_2_ on the membrane may cause profilin to switch between membrane-bound and actin-bound forms, making it an efficient mediator of external signals to microfilaments [49]. Twinfilin is a ubiquitous actin monomer-binding protein. Studies have found that in yeast and murine cells, twinfilin interacts with PtdIns(4,5)P_2_, and its actin monomer-sequestering activity is inhibited by PtdIns(4,5)P_2_ [50, 51]. Gelsolin, a severing and capping protein, inhibits further actin polymerization by capping the barbed ends of filaments [22, 52]. Interaction with PtdIns(4,5)P_2_ inhibits gelsolin’s severing activity, stabilizing the actin network [53–55]. Upon hydrolysis of PtdIns(4,5)P_2_, gelsolin becomes active, leading to filament severing and turnover.

PtdIns(4,5)P_2_ can also enhance de novo actin polymerization by modulating neural Wiskott–Aldrich Syndrome protein (N-WASP) activity, an essential regulator of actin dynamics. N-WASP is autoinhibited due to intramolecular interactions between its N-terminal and C-terminal regions, which mask the VCA (verprolin, central, acidic) domain—responsible for actin nucleation [56]. This autoinhibition is released when PtdIns(4,5)P_2_ and the small GTPase cell division control protein 42 (CDC42) simultaneously bind to N-WASP, leading to a conformational change that unmasks the VCA region [57, 58]. Once exposed, the VCA domain of N-WASP can bind to and activate the ARP2/3 complex, which promotes actin nucleation and polymerization [59]. PtdIns(4,5)P_2_ binding facilitates N-WASP activation and helps position N-WASP at membrane sites rich in actin nucleation activity, allowing for rapid and localized polymerization of actin filaments [59]. This coordinated regulation by PtdIns(4,5)P_2_ and CDC42 ensures precise spatiotemporal control of actin dynamics, particularly during cell movement, vesicle trafficking, and membrane protrusion formation [27, 60]. Through these interactions, PtdIns(4,5)P_2_ plays a pivotal role in reorganizing the actin cytoskeleton in response to extracellular signals.

PtdIns(3,4,5)P_3_, generated via class I phosphatidylinositol-3-kinase (PI3K)-mediated phosphorylation of PtdIns(4,5)P_2_, is a pivotal regulator of actin cytoskeletal dynamics, particularly in the processes of cell migration, signal transduction, and polarization [20, 61]. PtdIns(3,4,5)P_3_, accumulated at the membrane, creates a gradient that promotes the recruitment and activation of small GTPases such as Rac and CDC42, essential for initiating actin-rich structures like lamellipodia and filopodia, which drive directional movement [62–64]. Additionally, evidence suggests that PtdIns(3,4,5)P_3_ interacts with the WASP family verprolin homologous protein 2 (WAVE2) through its basic domain. WAVE2 is known to promote actin nucleation and branching, which are essential for the formation of dynamic actin networks [65–67]. The amino-terminal region of WAVE contains the PtdIns(3,4,5)P_3_-binding sequence, allowing PtdIns(3,4,5)P_3_ generated by PI3K at the cell membrane to recruit WAVE to polarized membrane regions directly [68–70]. This recruitment is critical for forming lamellipodia at the leading edge, an essential step in directed cell migration.

Like PtdIns(4,5)P_2_, PtdIns(3,4,5)P_3_ modulates ABPs, including profilin, by altering its interactions with actin monomers [48, 71]. The affinity of profilin for PtdIns(3,4,5)P_3_ is slightly higher than for PtdIns(4,5)P_2_, further influencing actin filament dynamics by shifting profilin from its monomer-sequestering function to actin polymerization [48]. Thus, PtdIns(3,4,5)P_3_ is a critical mediator linking external signals to cytoskeletal reorganization, supporting cell migration, polarization, and membrane trafficking.

PtdIns(3,4)P_2_ plays a critical role in regulating actin dynamics and adhesion processes, particularly in FA assembly [72]. Locally produced by enzymes such as class II PI3K and SHIP, PtdIns(3,4)P_2_ accumulates at specific subcellular sites to coordinate the recruitment of actin-binding and scaffolding proteins. In FAs, PtdIns(3,4)P_2_ generated by class II PI3K-C2β can promote FA disassembly [73]. PtdIns(3,4)P_2_ promotes RhoA-dependent stress fiber turnover by recruiting the PtdIns(3,4)P_2_-dependent RhoA-GTPase activating protein ARAP3. Furthermore, Studies have enriched the details of the molecular interactions of PtdIns(3,4)P_2_ during FA transformation [72, 74]. The PtdIns(3,4)P_2_, present in minimal amounts, mediates the recruitment of the Tks5-Grb2 scaffold, followed by N-WASP accumulation and podosome formation near newly formed FAs, affecting cell motility.

PtdIns(3,4)P_2_ regulates actin remodeling during phagocytosis by recruiting Lamellipodin (Lpd) to nascent phagosomes [75]. Lpd, in turn, organizes actin filaments via vasodilator-stimulated phosphoprotein (VASP). Depleting PtdIns(3,4)P_2_ disrupts pseudopod extension and blocks particle engulfment, underscoring its essential role in actin dynamics during phagosome formation [75].

By regulating these actin-associated proteins, PIP_n_ signaling maintains a critical balance between actin filament assembly and disassembly, governing essential cellular operations, including motility, morphological changes, and membrane trafficking. This regulatory mechanism is vital for the cell’s ability to respond to extracellular signals and maintain structural integrity. Overall, PIP_n_ signaling orchestrates the dynamic remodeling of the actin cytoskeleton, enabling fundamental activities like migration, division, and trafficking.

## PIP_n_ signaling at intermediate filaments (IFs)

**Fig. 3:**
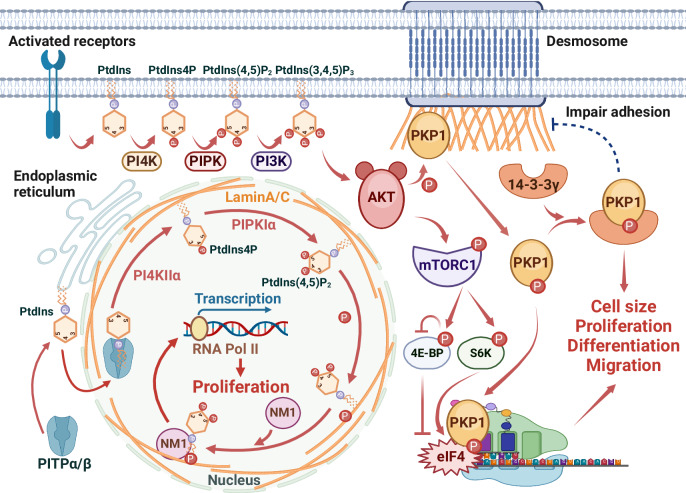
PIP_n_ signaling in IFs regulating cell growth and motility. This figure illustrates the pivotal role of PIP_n_ signaling in regulating cell proliferation and migration through its interaction with IFs. Activation of cell-surface receptors triggers the production of PIP_n_s (e.g., PtdIns4P, PtdIns(4,5)P_2_, PtdIns(3,4,5)P_3_), leading to AKT activation. This activation prompts AKT to phosphorylate PKP1, causing it to disengage from desmosomal IFs and diminishing cell adhesion. The phosphorylated PKP1 then interacts with 14-3-3γ, which fosters cell proliferation and migration. The stimulation of S6K and inhibition of 4E-BP by mTORC1-mediated phosphorylation downstream of the PI3K/AKT signaling pathway promote protein synthesis by influencing the eIF4 complex. The phosphorylation of PKP1 enhances eIF4 activity, which relaxes the complex structures in the 5’-UTR of mRNA, leading to increased protein production and cell growth. Within the nucleus, Lamin A/C interacts with PtdIns(4,5)P_2_, likely generated through the sequential actions of nuclear class I PITPα/β, PI4KIIα, and PIPKIα. The binding of PtdIns(4,5)P_2_ promotes the phosphorylation of Lamin A/C, leading to the formation of a complex with NM1. This complex activates RNA polymerase II (Pol II) transcription, thereby driving cell proliferation. This diagram is generated using BioRender.

IFs are a diverse assembly of protein fibers that form the cytoskeleton with microtubules and microfilaments. These filaments, composed of various proteins made up of amino acid chains, are characterized by a diameter of ~10 nm [76] and provide mechanical strength to cells and tissues, playing a crucial role in maintaining cellular integrity [77]. While microfilaments and microtubules are polymers of single types of proteins—actin and tubulin, respectively—IFs consist of a diverse array of proteins expressed across various cell types. Over 50 different IF proteins have been identified, and they are classified into six groups based on similarities in their amino acid sequences. IFs create an extensive network within the cytoplasm, extending from a ring that encircles the nucleus to the plasma membrane [78]. The maturation of IFs begins with the formation of parallel dimers, which associate in an anti-parallel, staggered manner to form tetramers, ultimately culminating in mature filaments consisting of about eight tetramers [79]. Known for their remarkable stability, IFs are vital for tensile strength, particularly in robust structures such as hair, scales, and fingernails [80]. PIP_n_ signaling is deeply involved in regulating cell growth, differentiation, survival, and migration [81]. These pathways are intricately linked to IFs, which, beyond being critical components of the cytoskeleton, also contribute to the modulation of cellular responses [82] (Fig. 3).

Keratins are a diverse family of IF proteins primarily found in epithelial cells [83]. They provide essential structural support, maintaining the mechanical integrity of cells and tissues. Keratin 8 (K8) and Keratin 18 (K18), often co-expressed, are crucial for the function of simple epithelial cells, including normal hepatocytes in the liver [83]. Beyond their structural role, these keratins are also involved in cellular signaling pathways. Although K8/18 expression persists during tumorigenesis, their loss during epithelial-mesenchymal transition (EMT) is linked to metastasis and chemoresistance [84]. K8/18 depletion leads to hyperactivation of the PI3K/AKT/ nuclear factor kappa B (NF-κB) pathway and increased expression of matrix metalloproteinase 2 (MMP2) and matrix metalloproteinase 9 (MMP9), which prompts the tight junction protein claudin-1 to assume an active role in cancer progression [84]. In addition, K8/18 directly interacts with AKT, facilitating reciprocal AKT hyperglycosylation and hypophosphorylation, reducing AKT kinase activity [85]. Keratin 10 (K10) has also been reported to interact directly with AKT [86]. While K10 is not a substrate of AKT, it binds to AKT through its N-terminal domain and inhibits cell proliferation and tumorigenesis by impeding AKT activity [86, 87]. Although it remains unclear whether Keratin 17 (K17) directly interacts with AKT, K17 does affect AKT activity [88, 89]. Reduced K17 expression results in decreased AKT and mechanistic target of rapamycin (mTOR) kinase activity, leading to a lower proliferation rate than normal K17 expression levels. The complex interplay between keratins and the PI3K/AKT pathway underscores the importance of these proteins in cellular regulation. Understanding how keratins modulate the PIP_n_ signaling could reveal new therapeutic targets for diseases characterized by aberrant keratin expression or AKT pathway activation, such as cancer.

Vimentin, a type III IF protein, maintains cellular structure and function. It self-assembles into a resilient cytoskeletal network composed of long, flexible filaments that preserve cell shape and resist mechanical stress, particularly in mesenchymal cells undergoing significant morphological changes [90, 91]. Vimentin also contributes to forming nuclear microdomains that facilitate gene expression and DNA repair [92]. Vimentin’s interaction with the PI3K/AKT pathway is significant in cancer progression and EMT [93]. Activation of growth factor receptors initiates a cascade that leads to vimentin phosphorylation at serine 39 by AKT. This promotes cell motility and invasion while protecting vimentin from caspase-mediated degradation, thereby enhancing cell survival [94, 95]. The phosphorylated vimentin also strengthens its binding to 14-3-3 proteins, inhibiting pro-apoptotic signaling pathways and contributing to chemoresistance in many cancer cells [96, 97]. During EMT, the hyperactivation of the PI3K/AKT pathway increases vimentin levels. It decreases the expression of epithelial markers like E-cadherin, fostering a mesenchymal phenotype characterized by increased motility and invasiveness. Vimentin’s interaction with mitogen-activated protein kinase kinase 4 (MAP2K4) propagates the effect of MAP2K4 on promoting breast cancer cell proliferation, migration, and invasion by activating the PI3K/AKT pathway, the downstream proteins such as c-JUN, the G1/S cell cycle, and the EMT [98]. Besides, patients with high expression levels of both MAP2K4 and vimentin exhibit poorer overall survival rates compared to those with low expression levels of these proteins [98].

Moreover, vimentin engages with the mTOR pathway, particularly mTORC1. Vimentin facilitates the activation of mTORC1 by modulating Rag GTPase activity, thereby enhancing ribosome biogenesis and protein synthesis, which is critical for supporting cell growth and survival [99]. This highlights vimentin’s potential as a therapeutic target for inhibiting cancer spread and improving patient outcomes. Further research is needed to elucidate how vimentin influences cancer biology and develop targeted therapies against it.

Lamin A/C belongs to the family of IF proteins known as nuclear lamins, located in the fibrous protein layer or network beneath the inner nuclear membrane of the cell nucleus [100]. They have a close structural relationship with the nuclear envelope, chromatin, and nuclear pore complexes. Lamin A and Lamin C are two different proteins produced by the same gene *LMNA* through alternative splicing [100, 101], and they play a crucial role in maintaining the structural stability of the cell nucleus, cell motility, mechanosensing, chromosome organization, gene regulation, cell differentiation, DNA damage repair, and telomere protection [102]. It functionally interacts with PtdIns(4,5)P_2_, a significant phospholipid involved in various cellular signaling processes, which is predominantly enriched in the nucleoplasm due to the orchestrated actions of class I PITPα/β, phosphatidylinositol-4-kinase II alpha (PI4KIIα), and phosphatidylinositol-4-phosphate 5-kinase type I alpha (PIPKIα) [9–11, 103].

Research has shown that Lamin A/C forms a complex with PtdIns(4,5)P_2_ and nuclear myosin I (NM1), with the assembly of this complex being influenced by Lamin A/C’s phosphorylation status; the formation of the complex facilitates the transcription of RNA polymerase II (Pol II), thereby promoting cell proliferation [103]. Besides PtdIns(4,5)P_2_, Lamin A/C is further linked to PIP_n_ signaling as the mechanistic study indicates that Lamin A/C-related malignant behavior is regulated by modulation of the PI3K/AKT/phosphatase and tensin homolog (PTEN) signaling pathway [104]. Lamin A/C knockdown or overexpression decreased or increased the protein levels, respectively, of the PI3K subunits p110 and p85 in prostate tumor cell lines, including lymph node carcinoma of the prostate (LNCaP), DU145, and prostate cancer cell line-3 (PC3). Furthermore, small hairpin RNA-mediated knockdown or overexpression of Lamin A/C resulted in the inhibition or stimulation of cell growth, colony formation, migration, and invasion, suggesting that Lamin A/C influences cell motility via PIP_n_ signaling [104].

In addition, the binding of Lamin A/C to PtdIns(4,5)P_2_ may help organize functional nuclear microdomains essential for gene expression and DNA repair [102, 103]. Furthermore, PI3K-C2β co-localizes with Lamin A/C in the nuclear matrix and is activated in the nuclear matrix [105, 106], suggesting the potential involvement of PtdIns3P and PtdIns(3,4)P_2_ in the functional role of nuclear lamina. These findings demonstrate a significant molecular link between PIP_n_s and nuclear IFs. It accentuates the essential role of Lamin A/C, transcending its traditional function of providing structural integrity.

Desmosomes are specialized cell adhesion junctions crucial for maintaining tissue integrity and withstanding mechanical stress, particularly in epithelial tissues [107–109]. They consist of transmembrane glycoproteins known as desmosomal cadherins, including desmogleins and desmocollins, which are connected to the IF system through a complex of proteins in the cytoplasmic plaque, such as desmoplakin, plakoglobin, and plakophilin. These proteins interact with the carboxyl-terminal domain of desmosomal cadherins, linking them to IFs like keratins [109]. Beyond their structural function, desmosomes are dynamic entities capable of influencing cell signaling pathways, including the modulation of proteins such as p38 mitogen-activated protein kinase (MAPK), which plays an integral role in migration and differentiation, among other processes [110, 111]. Their interactions with signaling molecules suggest that desmosomes contribute to cell behavior, vital in biological projects such as wound healing and tissue regeneration.

Upon stimulation by agonizts such as insulin, cytosolic PIP_n_s responsive to the cell surface receptors are produced, activating the downstream PI3K/AKT/mTOR/S6K signaling pathway, promoting cell proliferation, cell cycle progression [111]. Specifically, AKT2 phosphorylates desmosomal protein plakophilin 1 (PKP1), prompting its translocation from the cell membrane to the cytoplasm [112]. In the cytoplasm, phosphorylated PKP1 is stabilized and protected from degradation through binding with 14-3-3γ, leading to reduced intercellular adhesion but increased proliferation, migration, and anchorage-independent growth [113]. This phosphorylation event weakens PKP1 interactions with desmosomal proteins, such as desmoplakin (DSP) and desmoglein 1 (DSG1), thereby impairing intercellular adhesion. Further downstream, mTORC1 phosphorylates S6K and eukaryotic initiation factor 4E (eIF4E)-binding protein (4E-BP), resulting in the stimulation of S6K and inhibition of 4E-BP [111, 114]. This promotes the assembly of the eukaryotic initiation factor 4F (eIF4F) complex. Together, mTOR and S6K control the association of translation initiation factors with the eIF4 complex on mRNAs, thereby enhancing protein synthesis [111, 115]. Phosphorylated PKP1 interacts with this complex, stimulating eIF4 activity and facilitating the unwinding of secondary structures in the 5’untranslated region (5’-UTR), which correlates with increased protein biosynthesis, proliferation, and cell growth [115]. The connection between desmosomal proteins and the PI3K/AKT pathway suggests that desmosomes and their associated IFs may facilitate the transmission of signals from the cell membrane to the cytosol, influencing cellular activities such as proliferation and differentiation.

The intricate interplay between IFs, PIP_n_ signaling, and cellular adhesion structures such as desmosomes is essential for maintaining cellular and tissue integrity. IFs, known for their remarkable mechanical strength, are the backbone for cellular resilience, while PIP_n_ signaling pathways function as a regulatory hub, influencing growth, survival, and cellular behavior. Moreover, the dynamic nature of desmosomes not only ensures the structural stability of tissues but plays a crucial role in modulating cellular responses to external stimuli. Collectively, these components form a complex network fundamental to the health and functionality of cells and tissues, highlighting their importance in understanding and treating diseases where these systems are disrupted.

## PIP_n_ signaling at microtubules

Microtubules are cylindrical polymers composed of alpha- and beta-tubulin dimers that assemble into hollow tubes with a diameter of ~25 nm [116–118]. These highly dynamic structures exhibit intrinsic polarity, characterized by a fast-growing plus (+) end, where tubulin subunits are preferentially added, and a slower-growing minus (−) end, which is typically anchored to microtubule-organizing centers (MTOCs), such as centrosomes [116–118]. This polarity underpins many of the functions of microtubules, enabling directional transport and structural organization within the cell.

Microtubules are vital in resisting compressive forces within the cell, providing structural support, and maintaining cell shape, especially in cells with complex morphologies like neurons [118, 119]. In addition, they are essential in cellular processes such as intracellular transport and mitosis. During mitosis, microtubules form the mitotic spindle, a structure critical for segregating chromosomes into daughter cells, ensuring faithful cell division [116, 120]. Given their importance in maintaining cellular architecture, defects in microtubule function or regulation are associated with various diseases, including neurodegenerative disorders and cancers, highlighting the necessity of precise control over microtubule dynamics [121–123].

Microtubule-associated proteins (MAPs) are essential regulators of microtubule dynamics. MAPs stabilize microtubules by binding along their sides, reducing the depolymerization rate, or promoting polymerization at the plus end [124–127]. Other MAPs, such as kinesin and dynein, utilize microtubules as tracks for intracellular transport that direct cargoes like vesicles and organelles to specific locations within the cell [128–130]. For example, MAPs like tau stabilize long microtubule tracks in neurons, which are crucial for axonal transport. In contrast, in mitotic cells, MAPs, like the family of kinesin-related motor proteins, assist in forming the mitotic spindle, ensuring accurate chromosome segregation [131–134].

PIP_n_ signaling pathways are crucial for regulating microtubules, specifically in their stabilization and modulation through MAPs [14, 55, 124, 135, 136]. Specifically, PtdIns(4,5)P_2_ has been shown to inhibit microtubule assembly by directly and selectively binding to tubulin, a process that is not observed with other phospholipids like PtdIns(3,4,5)P_3_, PtdIns3P, Phosphatidylcholine (PC), Phosphatidylethanolamine (PE), or Inositol (1,4,5)-trisphosphate (Ins(1,4,5)P_3_) [135]. The reduced PtdIns(4,5)P_2_ levels can lead to the disorganization of microtubule networks, as seen in *Drosophila* male germ cells, demonstrating its regulatory importance [137].

**Fig. 4:**
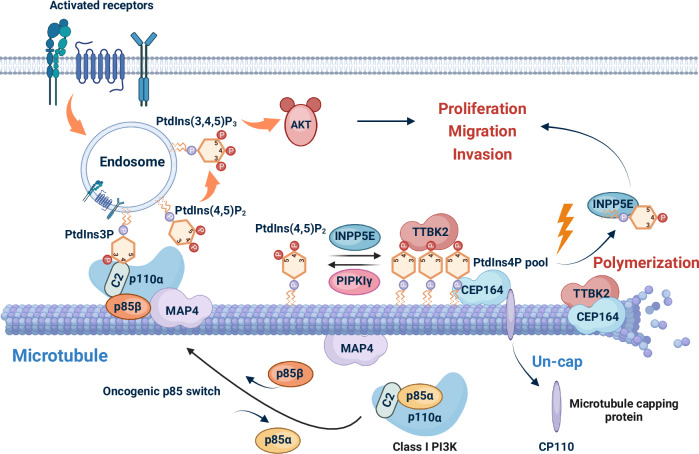
Role of PIP_n_ signaling in regulating microtubule dynamics. The schematic illustrates the interplay between activated receptors at the cell membrane, endocytosis, PIP_n_ signaling, and microtubule dynamics. Receptor activation generates various PIP_n_s (e.g., PtdIns3P, PtdIns(4,5)P_2_, PtdIns(3,4,5)P_3_) at endosomes, driving the recruitment and activation of AKT, which promotes cellular proliferation, migration, and invasion. Within this process, endosomal PtdIns3P recruits class I PI3K components (p110α/p85) by binding to the C2 domain of p110α. This complex is further linked to microtubules through interactions between MAP4 and PI3K. In cancer cells, the switch of the PI3K regulatory subunit from p85α to p85β enhances binding to PtdIns3P, amplifying complex recruitment and promoting constitutive PI3K/AKT signaling. At the microtubule-organizing centers (centrosomes), an accumulation of PtdIns4P occurs, where it interacts with TTBK2 and the accessory factor CEP164, preventing their interaction and inhibiting the removal of microtubule capping protein CP110 and microtubule extension. PIPKIγ reduces PtdIns4P levels by phosphorylating it to PtdIns(4,5)P_2_, which promotes TTBK2 recruitment and microtubule polymerization. In contrast, dephosphorylation of PtdIns(4,5)P_2_ back to PtdIns4P by INPP5E reverses this effect. This diagram is generated using BioRender.

Various PIP_n_s are modified by kinases and phosphatases, with several kinases implicated in microtubule homeostasis (Fig. 4). PI3K, for example, is associated with α/β-tubulin, and this interaction intensifies following insulin stimulation. Given that PtdIns(4,5)P_2_ is a primary substrate of PI3K, it likely plays a critical role in modulating microtubule responses to insulin signaling [136].

Phosphatidylinositol-5-phosphate 4-kinase type II gamma (PIP4KIIγ), an enzyme that synthesizes PtdIns(4,5)P_2_, plays a role in regulating microtubule dynamics. During mitosis, the loss of PIP4KIIγ increases the accumulation of mitotic centromere-associated kinesin (MCAK), a microtubule-depolymerizing kinesin, at spindle poles, leading to destabilization of spindle pole-associated microtubules [16]. Additionally, PtdIns(4,5)P_2_ binds to polo-like kinase 1 (PLK1), reducing PLK1-mediated MCAK phosphorylation—a modification that activates MCAK in depolymerizing spindle microtubules. These findings suggest that PIP4KIIγ and PtdIns(4,5)P_2_ contribute to microtubule stability by preventing depolymerization [16]. In contrast, another kinase that generates PtdIns(4,5)P_2_, PIPKIα [138], directly interacts with kinesin superfamily protein 2A (KIF2A), a member of the kinesin-13 family known for its microtubule-depolymerizing activity [139, 140]. This interaction enhances the microtubule-depolymerizing activity of KIF2A [140]. In neurons, PIPKIα suppresses the elongation of axon branches in a KIF2A-dependent manner [140], suggesting a unique PIPKIα-mediated mechanism that controls microtubule dynamics during neuronal development.

PIP_n_s engage with several MAPs to regulate microtubule dynamics, stability, and function. Studies from 1992 have demonstrated that PI can bind to MAP2 and inhibit microtubule assembly [124]. Our recent study has further revealed that PI3K binds to microtubules in a MAP4-dependent manner [14]. MAP4, a microtubule-associated protein, interacts with the C2 domain of the p110α catalytic subunit of PI3K through its microtubule-binding domain (MTBD). This interaction is essential for adequately functioning the PI3K pathway, which is often dysregulated in diseases such as cancer. The C2 domain of p110α is also known to bind with PtdIns3P enriched at the endosome surface [141], which is vital for the docking of class I PI3K to endosomes [142]. During oncogenesis, the regulatory subunit switch from p85α to p85β occurs in PI3K, ensuring induced activity [142]. These factors collectively contribute to the localization of class I PI3K to endosomal vesicles on microtubules, facilitating its association with receptor tyrosine kinases (RTKs). Activated RTKs are translocated to endosomes via endocytosis, where PI3K generates PtdIns(3,4,5)P_3_, activating the AKT signaling pathway and supporting microtubule homeostasis. As endosomes actively transport materials within the cell and facilitate their transfer to various compartments—such as the endoplasmic reticulum, mitochondria, the Golgi complex, and the nucleus—PIP_n_ signaling is vital for their movement along microtubules [143–147]. This process impacts cargo delivery to subcellular organelles and plays a significant role in cell motility and adhesion.

Tau, a MAP responsible for microtubule stabilization, can be phosphorylated by Tau Tubulin kinase 2 (TTBK2), a serine/threonine protein kinase of the Casein kinase 1 (CK1) superfamily [148, 149]. TTBK2 is vital for removing the centriolar coiled-coil protein of 110 kDa (CP110) and caps the distal end of the centriole, thereby facilitating microtubule elongation and ciliogenesis [148, 150–152]. A study revealed that PtdIns4P accumulates at the centromeres of non-ciliated cells, where it binds to TTBK2 and the distal accessory protein CEP164 (Centrosomal protein of 164 kDa) [153]. This interaction inhibits the recruitment of TTBK2, thus preventing CP110 removal. PIPKIγ reduces PtdIns4P levels, promoting TTBK2 recruitment and the initiation of ciliogenesis. Additionally, inositol polyphosphate-5-phosphatase E (INPP5E) inhibits the recruitment of TTBK2 and ciliogenesis by dephosphorylating PtdIns(4,5)P_2_ to PtdIns4P.

Additionally, PtdIns(3,4)P_2_ biosynthesis regulates integrin endocytosis and thus affects microtubule dynamics [154]. PtdIns(3,4)P_2_-rich microdomains at invadopodia guide integrin β3 endocytosis through SNX9-dependent membrane invagination and dynein-mediated retrograde transport, facilitating adhesion disassembly and recycling. Furthermore, PtdIns(3,4)P_2_, produced by SHIP1 and class II PI3K, recruits SNX9 and then facilitates vesicle trafficking during apical surface remodeling [155]. Perturbing PtdIns(3,4)P_2_ disrupts polarization by causing subcortical vesicle retention, implicating its role in coordinating microtubule-based transport of apical vesicles.

These regulatory mechanisms underscore the critical role of PIP_n_ signaling in maintaining microtubule integrity and ensuring proper cellular function across processes such as mitosis, intracellular trafficking, and cell migration. PIP_n_ signaling is crucial in preserving cellular architecture and function through microtubule dynamics and associated pathways.

## Discussion

**Fig. 5:**
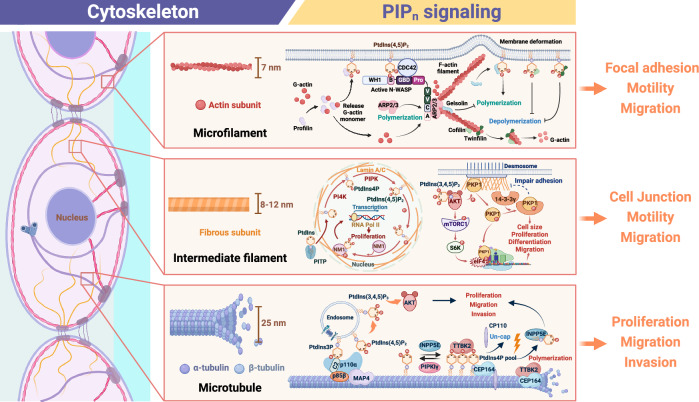
PIP_n_ signaling at the cytoskeleton in the regulation of cell dynamics. This figure provides an integrated overview of the regulatory mechanisms by which PIP_n_s influence the cytoskeleton, including the dynamics of microfilaments, IFs, and microtubules. It highlights the complex interactions between PIP_n_s, various effector proteins, and signaling pathways essential for cytoskeletal regulation, supporting critical cellular structures and processes such as FA, cell junction, proliferation, motility, migration, and invasion. This diagram is generated using BioRender.

PIP_n_ signaling elucidates critical roles in regulating the dynamics of various cytoskeletal components, including microfilaments, microtubules, and IFs (Fig. 5). The findings indicate that PIP_n_ signaling, mainly through PtdIns(4,5)P_2_ and PtdIns(3,4,5)P_3_, is essential for maintaining cytoskeletal integrity, facilitating intracellular transport, and enabling cell motility [156–158]. Specifically, PIP_n_ signaling interacts with a range of ABPs and MAPs, highlighting its role as a central regulatory mechanism in coordinating cytoskeletal dynamics [14, 16, 22, 23, 37, 38, 55, 124, 135].

PIP_n_ microdomains form at specific subcellular sites through localized activities of kinases and phosphatases. Class I and class II PI3Ks, alongside PIP_n_ phosphatases like SHIP1, regulate the accumulation of PIP_n_s at distinct cortical regions [14, 105, 106, 155]. These microdomains compartmentalize signaling, directing processes like actin remodeling and cellular polarization. The localization of PIP_n_-generating kinases is regulated by interactions with scaffold proteins, cytoskeletal elements, and pre-existing lipids. For instance, class II PI3K-C2β co-localizes with Lamin A/C in the nuclear matrix, while class I PI3K associates with microtubules through MAP4 [105, 106, 142]. These mechanisms ensure spatiotemporal control of cytoskeletal polymerization and dynamics.

Different PIP_n_s exhibit distinct roles in cytoskeletal regulation. Specificity in PIP_n_ signaling is achieved through subcellular localization, differential binding affinities, and pathway interactions. PtdIns(4,5)P_2_ interacts with ABPs like profilin and cofilin to regulate actin turnover, while PtdIns(3,4,5)P_3_ influences microtubule dynamics via AKT signaling [22, 23, 37, 38, 142]. PtdIns(3,4)P_2_ specializes in integrin trafficking and cellular polarization by recruiting proteins like SNX9, linking membrane dynamics to cytoskeletal organization [154, 155]. PtdIns(4,5)P_2_ may regulate vimentin via AKT, whereas PtdIns(3,4,5)P_3_ stabilizes microtubules through distinct gradients [93, 142]. These mechanisms fine-tune cytoskeletal dynamics in processes like cell migration, division, and intracellular transport.

PIP_n_ signaling not only influences cytoskeletal function but is also regulated by cytoskeletal components. IFs, such as keratins, vimentin, and lamin A/C, engage in reciprocal interactions with PIP_n_ signaling, particularly impacting pathways like PI3K/AKT. For instance, keratins regulate AKT activity: K8 or K18 depletion hyperactivates the PI3K/AKT pathway, driving metastasis, while K10 inhibits AKT activity, suppressing tumorigenesis [84, 86, 87]. Vimentin phosphorylation by AKT enhances motility, invasion, and chemoresistance, especially during EMT [94, 95], while its interaction with MAP2K4 further propagates PI3K/AKT signaling [98]. Lamin A/C links to PIP_n_ signaling in the nucleus, modulating PI3K/AKT activity and influencing proliferation and DNA repair [103, 104]. This bidirectional regulation underscores a feedback mechanism essential for maintaining cellular homeostasis.

The implications of these findings extend to various pathological conditions. Dysregulation of PIP_n_ signaling pathways can contribute to diseases such as cancer, neurodegenerative disorders, and autoimmune conditions, highlighting the importance of understanding these signaling networks in cellular behavior and disease pathology [159–163]. The interdependence of microfilaments, IFs, and microtubules, regulated by PIP_n_ signaling, is crucial for cellular functions and responses to environmental stimuli, including migration, division, and intracellular transport.

Despite advances in actin filament research, the current understanding of PIP_n_ signaling in cytoskeletal dynamics highlights significant gaps regarding direct interactions with IFs and microtubules. Existing studies largely focus on indirect regulation via pathways like PI3K/AKT, leaving the mechanisms of direct PIP_n_-IF/microtubule modulation unclear. Future research should explore the interactions between PIP_n_ signaling and IFs/microtubules, particularly their contributions to cytoskeletal stability and dynamics in various cellular contexts. Developing therapeutic strategies targeting PIP_n_ signaling pathways also presents a promising avenue for restoring cytoskeletal integrity and function in diseases characterized by dysregulation. For instance, microtubule-targeting agents like paclitaxel (Taxol) stabilize microtubules and treat various cancers. Alternatively, agents like vincristine disrupt microtubule dynamics yet still show therapeutic value in the context of some cancers [164–166]. Moreover, drugs that enhance or mimic PIP_n_ signaling, such as the PtdIns(3,4,5)P_3_ analogs, may offer novel therapeutic options in contexts where PIP_n_ signaling is compromised [167]. In neurodegenerative diseases, drugs such as latrunculin, which sequesters actin monomers and disrupts actin polymerization, may offer insights into treatments that target actin dynamics [168–171]. Additionally, the role of PtdIns(3,4)P_2_ in coordinating microtubule and actin dynamics warrants further investigation to better understand its contributions to intracellular transport and membrane remodeling.

In conclusion, PIP_n_ signaling is a fundamental regulator of cytoskeletal dynamics with significant implications for understanding cell biology and developing therapeutic interventions. Further investigation into these pathways and the clinical application of drugs targeting cytoskeletal stability will enhance our knowledge of cellular processes and inform strategies for addressing cytoskeletal-related diseases.
